# Supplementary material for: Chiral structures of electric polarization vectors quantified by X-ray resonant scattering
Source: Nat Commun. 2022 Apr 5;13:1769. doi: 10.1038/s41467-022-29359-5 (PMC8983710; doi:10.1038/s41467-022-29359-5)
Supplement: Supplementary file 1 — Supplementary Information [file 41467_2022_29359_MOESM1_ESM.pdf]

Supplementary Information

Chiral structures of electric polarization vectors quantified by X-ray resonant scattering

Kim *et al.*

Supplementary Note 1. Statistical fluctuation of polarization vectors

Since the vortex pairs are not perfectly repeated throughout the layer, we need to consider how random disorder will alter the scattering. The resonant scattering intensity considering the statistical fluctuations of the polarization vector can be expressed as follows from equation (10)

$$\langle I\rangle = \frac{1}{2}(1+P_L)(\langle|F_{\sigma'\sigma}|^2\rangle + \langle|F_{\pi'\sigma}|^2\rangle) + \frac{1}{2}(1-P_L)(\langle|F_{\pi'\pi}|^2\rangle + \langle|F_{\sigma'\pi}|^2\rangle)$$

$$+P_C Im\left(\langle F^*_{\sigma'\pi}F_{\sigma'\sigma}\rangle + \langle F^*_{\pi'\pi}F_{\pi'\sigma}\rangle\right) \quad \text{(S1)}$$

where <… > denotes a statistical average for random fluctuations of the polarization vector, and the average for structure factors can be written as

$$F^*_{\alpha\beta}F_{\gamma\omega} = \frac{1}{N^2}\sum_{n,n'} e^{i\vec{q}\cdot\left((\vec{L}_n-\vec{L}_{n'})+(\vec{r}_n-\vec{r}_{n'})\right)}(\vec{\alpha}\cdot\boldsymbol{T}^*_n\cdot\vec{\beta})(\vec{\gamma}\cdot\boldsymbol{T}_{n'}\cdot\vec{\omega}) \quad \text{(S2)}$$

$$\langle F^*_{\alpha\beta}F_{\gamma\omega}\rangle = \frac{1}{N^2}\left\langle\sum_{n,n'} e^{i\vec{q}\cdot\left((\vec{L}_n-\vec{L}_{n'})+(\vec{r}_n-\vec{r}_{n'})\right)} f^*_n f_{n'}\right\rangle \quad \text{(S3)}$$

where α, β, γ, and ω all denote X-ray polarization, and the scalar product related to the AT in the last part of equation (S2) is replaced by a scalar value f_n in equation (S3). Considering small fluctuations in the displacement vector, when expressed as $\vec{r}_n = \langle\vec{r}_n\rangle + \delta\vec{r}_n$, equation (S3) can be written as follows.

$$\langle F^*_{\alpha\beta}F_{\gamma\omega}\rangle = \frac{1}{N^2}\sum_{n,n'} e^{i\vec{q}\cdot\left((\vec{L}_n-\vec{L}_{n'})+(\langle\vec{r}_n\rangle-\langle\vec{r}_{n'}\rangle)\right)} f^*_n f_{n'}\left\langle e^{i\vec{q}\cdot(\delta\vec{r}_n-\delta\vec{r}_{n'})}\right\rangle. \quad \text{(S4)}$$

Since $\delta\vec{r}_n$ implies a small change in the rotation angle $\phi_{n,i}$, the small fluctuation effect caused by $\delta\vec{r}_n$

can be included in $f_n^* f_{n'}$ containing the ATs. However, if $f_n^* f_{n'}$ is expanded for $\delta\vec{r}_n$ and high order terms are ignored, the small fluctuation of $f_n^* f_{n'}$ can be ignored because $\langle\delta\vec{r}_n\rangle$ is zero. Therefore, as in equation (S4), the average value is taken only in the phase term.

Now, to calculate the mean value in equation (S4), we use the relationship $\langle XY\rangle = \mathrm{cov}(X, \mathrm{Y}) + \langle X\rangle\langle Y\rangle$ for the variance, where $\mathrm{cov}(x, \mathrm{y})$ denotes a covariance of variables *x* and *y*, and the Baker-Hausdorff theorem for variables with a Gaussian probability distribution.[S1]

$$\left\langle e^{i\vec{q}\cdot(\delta\vec{r}_n - \delta\vec{r}_{n'})}\right\rangle = e^{-q^2\langle\delta r^2\rangle} e^{q^2\,\mathrm{cov}(\delta r_n, \delta r_{n'})} \qquad \text{(S5)}$$

Considering that small fluctuations of the polarization vector are isotropic regardless of the component in a specific direction, and $\langle\delta r_n^2\rangle = \langle\delta r^2\rangle$. As the distance between the unit cells of resonant ions increases, the covariance between them decreases. Therefore, the covariance between the polarization vectors of unit cell *n* and n' can be expressed as $\langle\delta r^2\rangle\,\exp(-\frac{|n-n'|}{\xi_c})$ by introducing the coherence length ξ_c in unit cells. As a result, equation (S4) is expressed as

$$\left\langle F_{\alpha\beta}^* F_{\gamma\omega}\right\rangle = \frac{e^{-q^2\langle\delta r^2\rangle}}{N^2}\sum_{n,n'} e^{i\vec{q}\cdot\left((\vec{L}_n - \vec{L}_{n'}) + (\langle\vec{r}_n\rangle - \langle\vec{r}_{n'}\rangle)\right)} f_n^* f_{n'}\, e^{q^2\langle\delta r^2\rangle} e^{-\frac{|n-n'|}{\xi_c}} \qquad \text{(S6)}$$

The above equation clearly shows that the effect of small fluctuations in polarization vectors is mainly represented by the coherence length ξ_c. As the coherence length ξ_c decreases, the contribution of cross terms with $n \neq n'$ decreases, so in the q_z-dependent AR curve, which will be described later, as q_z increases, the amplitude of oscillation decreases and broadens.

Supplementary Note 2. Effective penetration depth in soft x-ray resonant scattering

The electric-field intensity distributions as a function of incident angle and depth obtained for non-resonant (450 eV) and resonant (456.8 eV) energies were calculated using dynamical calculation,[S2] As shown in the left panel in Supplementary Fig. 1, a distinct total thickness fringe can be seen in the specular reflectivity at non-resonant energy (450 eV) away from the absorption edge. Also, in the q_z region where the experimental data was measured, it can be seen that the electric field intensities in the PTO/STO superlattice are 10% or more of the incident beam. On the other hand, in the 456.8 eV (right panel) where the resonant scattering experiment (Figs. 2d and 4m) was performed, the total thickness fringe begins to be seen only in the q_z region greater than 0.2 $Å^{-1}$. Convolution by angular divergence of 0.05 degree and interfacial roughness of 4 Å were also considered. In the case of the x-ray absorption spectroscopy (XAS) experiment, it is difficult to measure the part deeper than the penetration depth, where the x-ray intensity is reduced to 1/e, because the contribution to the XAS decreases rapidly. On the other hand, in the case of elastic X-ray scattering, the contribution of a region deeper than the penetration depth cannot be simply ignored because of the interference effect. For example, for the resonant energy (right panel), electric field intensities higher than 50% (~1/e) of the incident beam are confined to a fire or one or two $PbTiO_3$ layers close to the surface. Nevertheless, a total thickness fringe corresponding to eight bilayers can be observed in the specular reflectivity. Therefore, in soft x-ray scattering, unlike x-ray absorption spectroscopy, the maximum depth at which interference can occur may be deeper than the simple penetration depth.

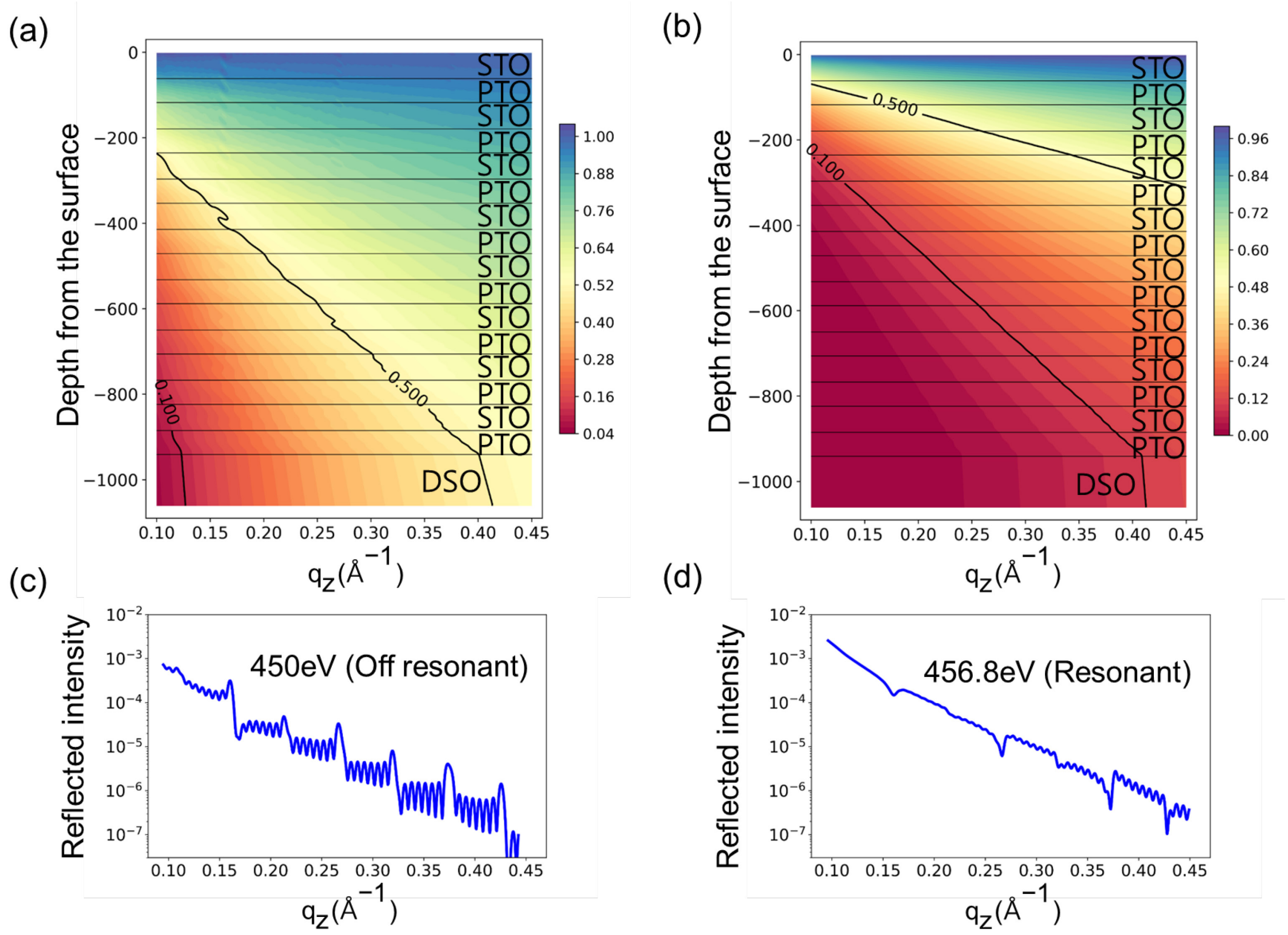

Supplementary Fig. 1 Electric-field intensity distributions (upper panel) and soft x-ray reflectivity curves (lower panel) calculated for non-resonant (left panel) and resonant energies around Ti L3 edge (right panel).

Supplementary Note 3. Asymmetry ratios for staggered vortex pairs

Let us take a closer look at resonant scattering for vortex pairs with opposite rotational directions that form a polar vortex array. First, the AR curves for the case where there is no difference in the depth direction of the vortex core positions (Fig. 4b) and the case where there is a difference by 2 unit cells (Fig. 4d) are very different from each other. (Figs. 4h and 4j) In order to understand the difference between the two cases, we calculated and compared cases where the difference in vortex core position was relatively small. In the case where the vortex core position difference is 0.5 unit cell (Supplementary Fig. 2b), some antisymmetric feature is seen between the AR curves of the satellites with opposite signs, but the AR curve itself is very different from the case where the vortex core positions match (Fig. 4b). Therefore, the AR curve changes very sensitively even if the difference in the position of the vortex core of the vortex pair is small within one unit cell. On the other hand, when the vortex core position difference is one unit cell (Supplementary Fig. 2c), the AR curves already show the same sign in most of the q_z region and are similar to those for the 2-unit-cell difference (Fig. 4d).

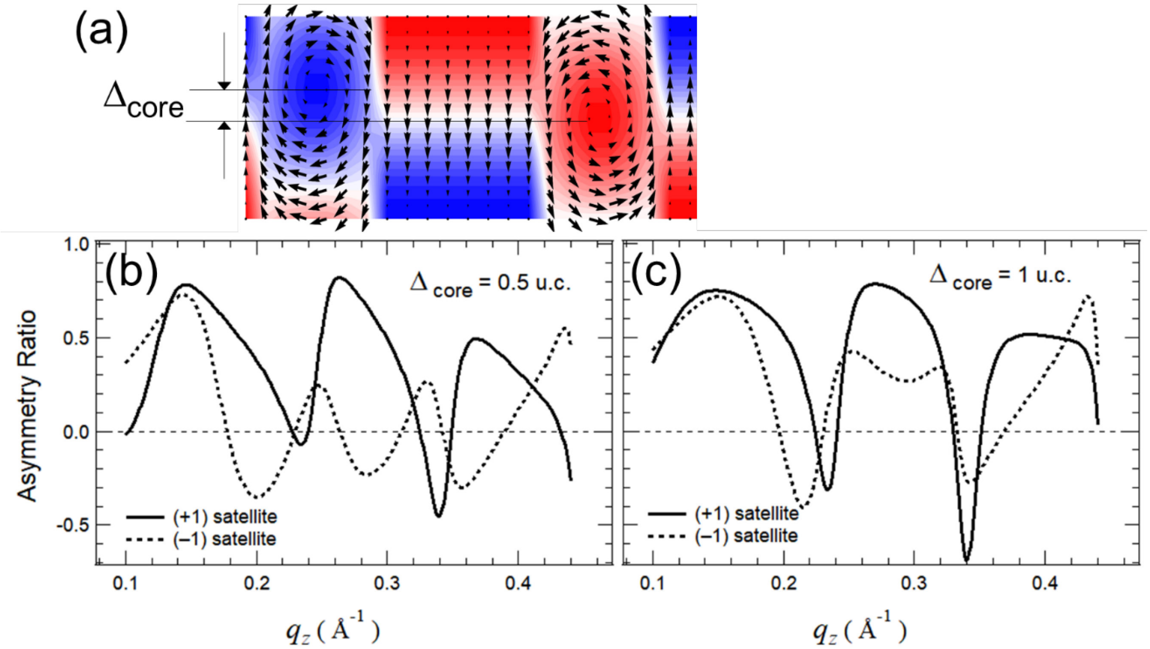

Supplementary Fig. 2 (a) polar vortex pair whose core positions differ by $\boldsymbol{\Delta_{core}}$. (b)-(c) Asymmetry ratios calculated for $\boldsymbol{\Delta_{core}}$ = 0.5 and 1 unit cells.

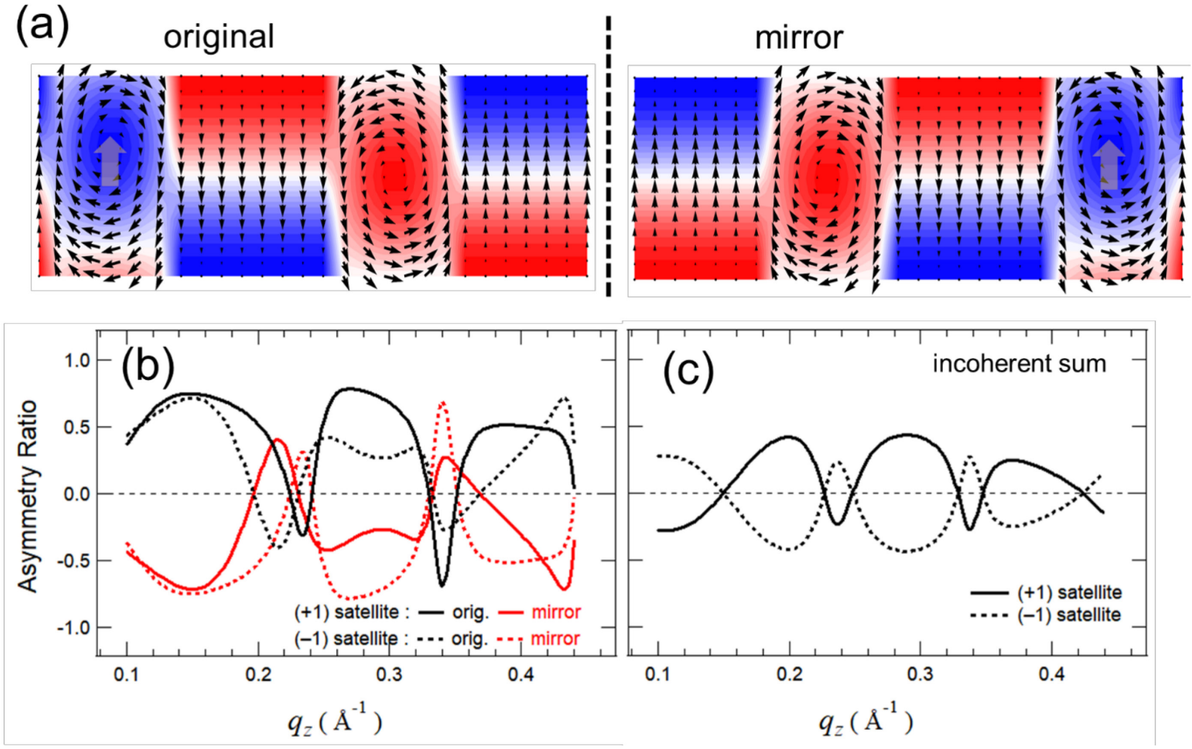

Supplementary Fig. 3 (a) A polar vortex array in which the core position of the vortex pair is shifted by one unit cell and a mirror-reflected image along the lateral direction. (b) Asymmetry ratios for original and mirrored vortex arrays. (c) Asymmetry ratios for incoherent sum of resonant scattering intensities from both original and mirrored vortex arrays, always showing an antisymmetry between those at opposite satellites.

References

S1. Als-Nielsen, J. & McMorrow, D. *Elements of Modern X-ray Physics*. (Wiley, 2011). doi:10.1002/9781119998365.

S2. Parratt, L. G. Surface Studies of Solids by Total Reflection of X-Rays. *Phys. Rev.* **95**, 359–369 (1954).
